# [Retracted] Effect of SATB1 silencing on the proliferation, invasion and apoptosis of TE‑1 esophageal cancer cells

**DOI:** 10.3892/ol.2025.15052

**Published:** 2025-04-22

**Authors:** Bo Huang, Fei Xiong, Siwang Wang, Xianping Lang, Xiaodong Wang, Hongli Zhou

Oncol Lett 13: 2915–2920, 2017; DOI: 10.3892/ol.2017.5854

Following the publication of the above paper, it was drawn to the Editor's attention by a concerned reader that, regarding three of the flow cytometric plots shown in Fig. 4 on p. 2919 (namely, Fig. 4B, C and D), these plots appeared to show similar groupings of dots, which would not have been anticipated if these experiments had been performed discretely under different experimental conditions, suggesting a fundamental flaw either in the way in which these experiments were performed, or in how the results were outputted.

The Editor of *Oncology Letters* has decided that this paper should be retracted from the Journal on account of a lack of confidence in the presented data. The authors were asked for an explanation to account for these concerns, but the Editorial Office did not receive a reply. The Editor apologizes to the readership for any inconvenience caused.

